# Selection of Specific Protein Binders for Pre-Defined Targets from an Optimized Library of Artificial Helicoidal Repeat Proteins (alphaRep)

**DOI:** 10.1371/journal.pone.0071512

**Published:** 2013-08-27

**Authors:** Asma Guellouz, Marie Valerio-Lepiniec, Agathe Urvoas, Anne Chevrel, Marc Graille, Zaineb Fourati-Kammoun, Michel Desmadril, Herman van Tilbeurgh, Philippe Minard

**Affiliations:** 1 Institut de Biochimie et Biophysique Moléculaire et Cellulaire, Université Paris-Sud, Orsay, France; 2 Unité Mixte de Recherche 8619, Centre National de Recherche Scientifique, Orsay, France; Center for Genomic Regulation, Spain

## Abstract

We previously designed a new family of artificial proteins named αRep based on a subgroup of thermostable helicoidal HEAT-like repeats. We have now assembled a large optimized αRep library. In this library, the side chains at each variable position are not fully randomized but instead encoded by a distribution of codons based on the natural frequency of side chains of the natural repeats family. The library construction is based on a polymerization of micro-genes and therefore results in a distribution of proteins with a variable number of repeats. We improved the library construction process using a “filtration” procedure to retain only fully coding modules that were recombined to recreate sequence diversity. The final library named Lib2.1 contains 1.7×10^9^ independent clones. Here, we used phage display to select, from the previously described library or from the new library, new specific αRep proteins binding to four different non-related predefined protein targets. Specific binders were selected in each case. The results show that binders with various sizes are selected including relatively long sequences, with up to 7 repeats. ITC-measured affinities vary with K_d_ values ranging from micromolar to nanomolar ranges. The formation of complexes is associated with a significant thermal stabilization of the bound target protein. The crystal structures of two complexes between αRep and their cognate targets were solved and show that the new interfaces are established by the variable surfaces of the repeated modules, as well by the variable N-cap residues. These results suggest that αRep library is a new and versatile source of tight and specific binding proteins with favorable biophysical properties.

## Introduction

It is now possible to create artificial proteins endowed with specific binding properties using combinatorial biology or directed evolution methods. The first step of this process is to identify a suitable protein scaffold and a set of side chains located on its surface that could be randomly substituted in order to create a binding site. A large library of variants of this scaffold is then constructed. The very few proteins of the library whose side chain combination create a specific binding site for a predefined protein target can be selected out from the library and amplified by phage, ribosome or cell display.

The scaffold protein has to respond to a number of criteria. An Ideal protein scaffold candidate should be sufficiently stable to tolerate a wide range of side chains combination on its binding surface [1]. As Ineffectiveness for intracellular applications as well as for prokaryotic expression are clear limits of most natural antibodies, a scaffold candidate should not be submitted to these restrictions. These artificial proteins must therefore be disulfide-free. In contrast to most natural antibodies, these artificial proteins could then be used as intracellular agents because their structure does not depend on disulphide bond formation. Finally a low susceptibility to aggregation is essential to allow further exploitation of scaffolds as modules in more elaborate multidomain construction [2] and as tools for structural studies [3].

A number of scaffold candidates have been proposed (see references [4]–[7] for comprehensive reviews) and new candidates were recently added to this list [8]–[13]. Despite the existence of a wide range of scaffolds, only few libraries yielded tight and specific binders against a wide panel of protein targets. Four scaffolds (affibodies [14], Adnectin [15]/monobodies [16], anticalins [17] and DARPins [18]) have produced many specific binders, some of which are being evaluated in clinical trials. A few others [19]–[22] more recently developed have also produced a range of specific binders.

A specific and promising class of protein scaffolds is based on natural protein repeat architectures such as ankyrin, leucine rich repeats (LRR) and tetratricopeptides (TPR) [23], [24]. In these natural protein families, the juxtaposition of variable side chains from consecutive repeats can create an extended binding surface without compromising fold stability. These protein architectures are versatile scaffolds for protein recognition and have often been recruited along cellular evolution for protein/protein recognition tasks.

The sequential determinants of a repeat architecture can be identified by analysis of sequence/structure correlations observed in a natural repeat family. If the sequence features are correctly extracted, it becomes possible to design new “idealized” repeat modules. The design of artificial repeats is however a demanding test since natural sequences families are often heterogeneous. Furthermore, due to the repeated character of the sequence, the incorporation of incorrect sequence features in the design can have cumulative effects on protein stability. In favorable cases, these self-compatible modules can be concatenated and inserted between appropriate capping modules to give rise to correctly-folded and very stable protein families. For example, designed ankyrin repeat proteins (DARPins) made by concatenation of idealized ankyrin repeats were shown to be efficiently expressed, well-folded and stable, independently of the precise side chain composition of the binding surface. Similarly, proteins made from LRR [25], Armadillo [26] and TPR [27] repeats have also been produced and shown to be folded [28].

Artificial repeat proteins can provide new binding functions: a range of proteins with new and specific properties have been selected from large DARPin libraries [18], [24]. TPRs were carefully engineered to provide peptide binders useful for cellular applications [29]. Proteins based on Armadillo repeats have recently been developed to select protein binding to specific peptide sequences [30]. Artificial proteins engineered from the LRR motif from the non-IG based antibodies of jawless vertebrates have recently been described. Members of this protein family named “repebody” were successfully engineered for tailored binding to targets [31].

We have recently reported the design of a new artificial repeat protein family named αRep. This design was based on a specific subgroup of helicoidal repeats commonly found in thermophilic microorganisms. Artificial proteins based on this design were shown to be well-expressed, folded as expected, and extremely stable. Synthetic procedures allowing the constitution of large phage display libraries based on αRep design were explored and a first generation library (hereafter referred to as Lib1.0) was described [32]. It was not known, however, whether or not libraries based on this repeat design could give rise to specific binders of pre-defined protein targets. We have now explored in detail this potential application of the αRep design.

In this paper, we show that the previously described αRep library, as well as a new improved version, can be successfully used to generate tight and specific binders against a range of pre-defined protein targets. In order to provide a structural basis for this molecular recognition process, we determined the crystal structures of two different protein complexes between αRep and their cognate targets.

## Results

A first αRep library (Lib1.0) was previously described and was used for selections of specific binders as described below. However this library was not fully optimized and at least two of its characteristics could be improved: first, the diversity of side chains in the randomized positions was introduced by a single oligonucleotide encoding all variable codons. To avoid proline and cysteine, a limited set of partially randomized codons was used and this simplified coding scheme resulted in an arbitrarily biased side chains distribution in the randomized positions. We have now created a new library (Lib2.0), in which the distribution of side chains was adjusted to mimic the side chains distribution observed in each position of natural repeats in this protein family.

The second and main default of the first library (Lib1.0) is due to the presence of a significant proportion (30%) of non-coding modules containing frame shift errors, resulting from incorrectly synthetized or miss-assembled oligonucleotides. The same default was found in the new library Lib2.0 with 20% of non-coding modules in spite of the new circle assembly procedure (Table 1). In those libraries, the number of repeats varies from protein to protein and longer sequences have increased probabilities of incorporating modules with sequence errors. Consequently, the vast majority of the correct αRep clones encode for relatively short proteins made of 3 or less internal motifs. The presence of potentially extended binding surface is an essential feature of natural repeat proteins and therefore one of our objectives was to improve the previously described library in order to sample more efficiently a diverse set of proteins with extended binding surfaces.

**Table 1:** Characteristics of the initial and optimized libraries.

Library	Library size	Proportion of correct motifs	In-frame clones^d^	In-frame clones (n≥1)^e^	Average motifs number^f^	Average motifs number (n≥1)^g^
Lib1.0^a^	3×10^8^	68%(77/112)	63%(31/49)	26%(13/49)	0.97±1.4	2.0±1.6
Lib2.0^b^	2.7×10^7^	80%(21/26)	74% (17/23)	30%(7/23)	0.8±1.1	2.0±1.2
Lib2.1^c^	1.7×10^9^	97%(94/97)	93% (41/44)	77%(34/44)	2.3±1.9	2.8±3.2

a Lib1.0 was constructed using single stranded circles for the RCA amplification.

b Lib2.0 was constructed using double stranded circles for the RCA amplification.

c Lib2.1 was constructed from Lib2.0 in two steps. First, in-frame sequences were recovered by filtration of the phage library on an anti-Flag antibody; second, the filtrated library was shuffled using only modules preselected to be in frame. The total number of clones for each library is indicated in the library size column.

d, e The ratio of coding sequences was determined from the sequence of randomly picked clones as the number of correct sequences versus the total number of sequences. “In-frame clones” (e) include all coding sequences while “in-frame clones (n ≥ 1)” (f) include coding sequences with at least one motif between the N-cap and the C-cap.

f, g The average number of motifs was calculated as the mean of inserted motifs in all coding sequences (g) or in all coding sequences with at least one motif between the N-cap and the C-cap (h).

The proportion of fully correct coding modules was improved by modifications in the library construction procedures. A new library was assembled by a two-step approach: a pool of correct modules from Lib2.0 was first pre-selected and then recombined to create the final library. The final optimized library (Lib2.1) contains a significant proportion of correct long αRep sequences (Table 1). The overall procedure is described below.

### Library 2.1 Design and construction

**Figure 1:**
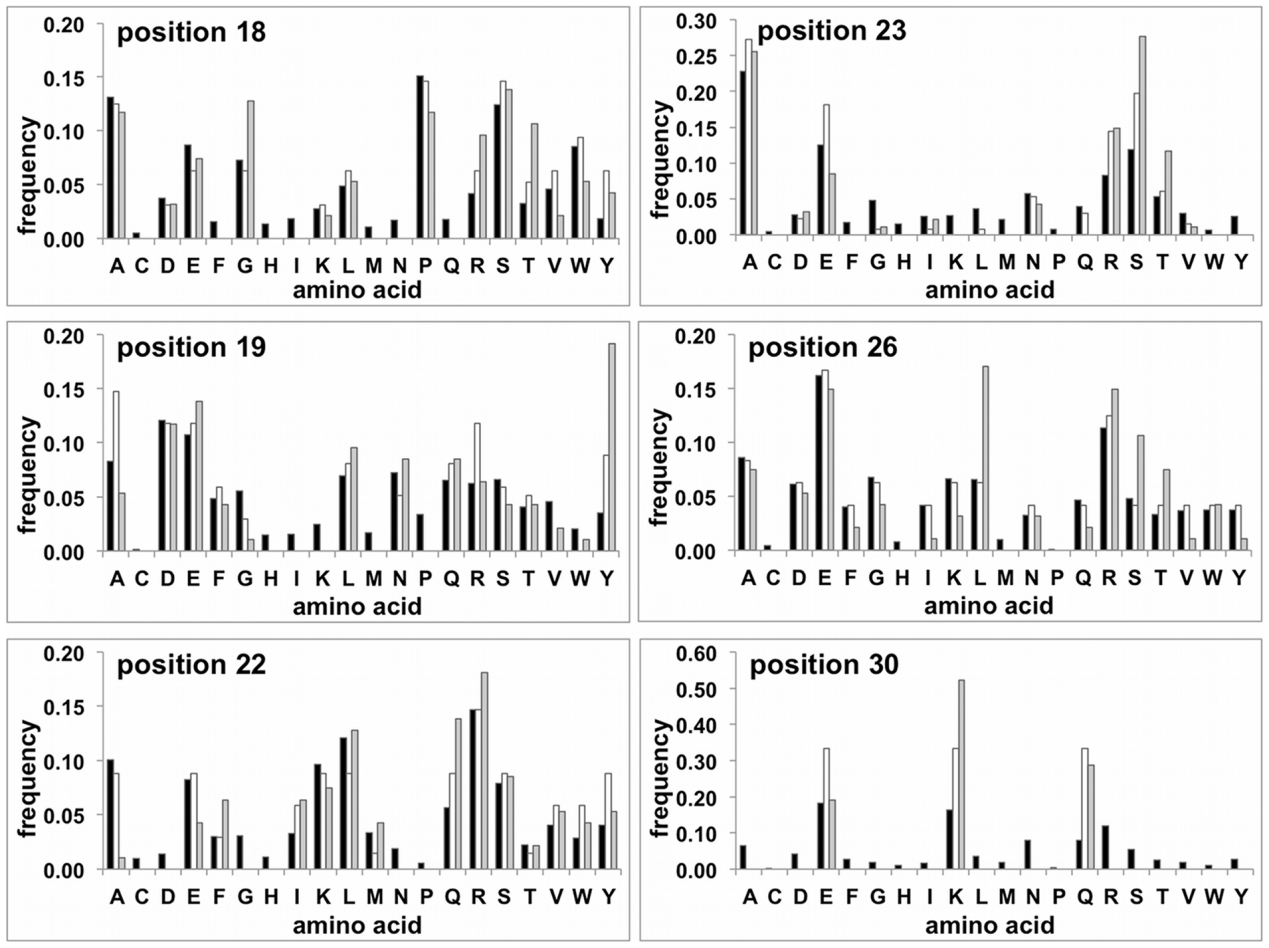
Sequence diversity of the optimized library (Lib2.1). The experimental amino acids frequency at each of the 6 randomized positions as compared to the natural and encoded frequencies. Black bars: Amino acid frequencies calculated from the natural collection of αRep like repeats; White bars Frequencies: expected from the coding scheme; Grey bars: experimental diversity observed in library Lib2.1.

In a first step, a new “primary” library (Lib2.0) was constructed using an improved randomization scheme. The diversity was encoded by a set of oligonucleotide cassettes, which were added in proportions calculated to match the targeted amino acid distribution. For each randomized position, the side chain diversity was encoded to mimic the natural distribution of side chains observed in a collection of natural αRep-like motifs. The underlying assumption is that the natural distribution of side chains at each variable position presumably results from a compromise between compatibility of each side chain within its structural context and its propensity to be involved in protein/protein interactions. The natural distribution of side chains cannot be defined globally for all repeat positions as it is clearly position dependent (Fig. 1): for example, proline is the most common residue at position 18 while it is absent at position 22 to 30. Other position specific biases are clearly apparent, such as the high frequency of small side chains at position 23, probably related to steric hindrance between neighboring αRep modules. The targeted distribution can simply be encoded by a set of oligonucleotides at position 26. However, for the two pairs of contiguous variable positions (18–19 and 22–23), the two vicinal randomized codons must be located on the same oligonucleotide. Therefore, encoding all of the 18–19 and 22–23 dipeptides would require a large number of oligonucleotides to match all possible amino acids combinations. To limit the number of oligonucleotides, the 18–19 dipeptide diversity was encoded by set of 26 partially degenerated cassettes, and similarly the 22–23 dipeptide diversity was encoded by 24 partially degenerated cassettes. This simplification implies that the combinatorial dipeptide diversity is only partially sampled: the degenerated cassettes encode 87 different dipeptides for the pair 18–19 and 60 dipeptides for the pair 22–23. The degenerated sequence cassettes were selected in such a way that most of the dipeptides encoded with this coding scheme are among the most frequent dipeptides in the natural sequence collection: 65 of the 87 encoded dipeptides 18–19 are among the 100 most common natural dipeptides and similarly 54 of the 60 encoded 22–23 dipeptides are among the 100 most common naturally observed dipeptides. Technical constraints were present at position 30 as this codon overlaps with nucleotides of the restriction site used for module assembly and for this reason cannot be freely modified. In the resulting library only three types of residues (Glu, Lys, Gln) are encoded at position 30. This limitation was found acceptable as the side chain distribution at this position is also naturally biased toward large polar side chains. Cysteine residue was systematically excluded from the coding scheme, at all positions.

This coding scheme encodes for approximately 2.5×10^5^ different repeat sequences (87×60×16×3). This ensemble of encoded repeats can be exhaustively sampled in an experimental library. However, the sequence space of αRep proteins made from several repeats is further increased by combinatorial repeats assembly and is therefore much larger than experimental libraries.

The N-cap module is structurally close to the repeated αRep modules and therefore the residues corresponding to the variable positions were also randomized. However a simpler diversification scheme was used: the N-cap codon corresponding to positions 18 and 19 was encoded using a partially degenerated codon vnk (encoding all amino acids except Cys, Trp, Tyr, Phe) and N-cap positions corresponding to 22, 23 and 26 were encoded by dht (encoding Ala, Asp, Glu, Phe, Ile, Lys, Leu, Met, Asn, Ser, Thr, Val, Tyr). These codons were chosen so as to introduce a relative diversity in these positions while still avoiding cysteine residues in all positions and proline residues in position 22, 23, 26 (Fig. 1).

### Construction of an optimized αRep library

The double-stranded oligonucleotide cassettes corresponding to degenerated repeat sequences were ligated together to produce double-stranded circular sequences corresponding to one repeat. The circular sequences were amplified by rolling circular amplification (RCA) and assembled, as previously described, to generate a library with a variable number of repeats. A library containing 2.7×10^7^ independent clones was obtained and named Lib2.0.

A set of randomly picked clones was used for sequence analysis. The 26 sequences obtained from Lib2.0 indicated that 80% of motifs had expected sequences while the remaining 20% encoded frame-shifted modules. Because of this proportion of incorrect modules the majority of correct sequences remained relatively short and the average size for in-frame clones was close to 2 (Table 1).

In a second step, the subset of modules from Lib2.0 encoding correct αRep proteins were recovered and recombined to generate the final library (Lib2.1). Briefly, correctly encoded proteins from Lib2.0 were displayed on the surface of the M13 bacteriophage with an N-terminal Flag-tag. In order to display a selectable Flag-tag, each phage clone must have an open reading frame covering the full protein sequence, from the export sequence up to the phage anchoring pIII-C terminal domain. At this step, phages produced from Lib2.0 were captured with an anti-Flag-tag antibody and amplified. This “filtrated” library contains only the subpopulation of Lib2.0 with a fully coding αRep nucleotide sequence. The repeats pool from this subpopulation was recovered and recombined by a module shuffling procedure to generate the final library (Lib2.1).

The resulting library, Lib 2.1, contained 1.7×10^9^ independent clones. A series of 44 sequences from randomly picked individual clones was analyzed and compared to the initial library (Lib1.0) and the primary library (Lib 2.0) (Table 1). Almost all clones from this shuffled library had a fully coding sequence. The sequences of the individual motifs were analyzed and the distribution of amino acids is shown in Figure 1. For the six randomized positions the experimental diversity was close to the targeted diversity, however with some relative deviations like over-representations of Gly18, Tyr19, Ser23 and Leu26. The origin of these biases is unknown and may result either from a biased incorporation of oligonucleotides at the library assembly stage, or from an uneven amplification of sequences during the module shuffling procedures.

Approximately one fourth of this library contained clones with no inserted modules in between N- and C-caps. Altogether, the useful part of this library, containing at least one internal motif and a fully correct sequence, was improved from 26% (13/49 clones) in Lib1.0 to 77% (34/44 clones) in Lib2.1. In addition, the size distribution was enlarged as a significant fraction of clones (8/44) contained a correct sequence and more than 5 internal motifs while such long coding sequences were not observed in samples of Lib1.0 and Lib2.0.

To summarize, compared to the previously described library [32] (Lib1.0), the new αRep library (Lib2.1) is more diverse and samples preferentially naturally frequent side chains. It is further enriched in coding sequences and consequently includes a higher proportion of longer proteins, with three or more internal motifs.

### Selection for specific binders of protein targets

At this step it was essential to test if the αRep libraries could efficiently give rise to αRep variants with tight and specific binding properties for predefined targets. Phage display selections were performed on a range of unrelated protein targets with different topologies. The proteins used as targets are described below.

Target A3 is an αRep protein randomly picked from Lib1.0, previously reported as αRep-N4–a [32]. Its biophysical properties and crystallographic structure were described. This protein forms a homodimer in solution. Each monomer contains four internal repeats plus the N- and C-caps, each one made by two antiparallel helices. With this protein target, a putative binding surface arises from helices juxtaposition.

Green fluorescent protein (EGFP) was used as an example of a β-barrel target and was retained to test if the αRep fold could bind non-helicoidal proteins. Furthermore, as GFP fusions are widely used for cellular applications, EGFP binders could be potentially useful as routine research reagents.

Target NCS-3.24 is a β-sandwich protein. Additionally, this protein is a variant of a bacterial protein, neocarzinostatin (NCS) with a limited set of substitutions previously engineered in one crevice of the protein [33]. Rising binders for the mutated form and testing specificity could therefore make a good test for the ability of αRep binders to find binding determinants in a concave surface as well as to discriminate between two related proteins.

The last target (Ebs1) was chosen as an example of a larger protein predicted to be an all-α protein, which functions in mRNA surveillance pathway in *S. cerevisiae* [34]; [35].

Phage display selections were performed on those four targets using different libraries and selection procedures. Lib2.1 was used for the selections against the targets EGFP and Ebs1; Lib1.0 was used with the targets A3 and NCS-3.24. The intermediate library Lib2.0 was also tested in a selection process against A3 (Table 2).

**Table 2:** Selection of binders from the αRep libraries.

Target	Target MW (kDa)	Target structure	Library^a^	Phage-ELISA screening^b^	Distinct sequences^c^	Soluble expression^d^	Clones used for further characterization^e^
A3	22.2	All-α	1.0	27% (23/84)	4/4	4/4	bA3-2 (1) bA3–1 (3)
A3			2.0	51% (43/84)	1/4	1/1	bA3-17 (2)
NCS-3.24	13.3	β-sandwich	1.0	3% (1/36)	1/1	1/1	bNCS-16 (2)
EGFP	31.6	β-barrel	2.1	12,9%(16/124)	3/14	3/3	bEGFP-A (6)
Ebs1	70.8	All-α	2.1	32% (23/72)	9/9	8/8	bEbs1-6 (7)

a The libraries indicated were used for the selections against the corresponding targets.

b The phage-ELISA results are indicated as the number of clones giving a positive signal *versus* the number of clones tested. A Clone was scored as positive if its measured signal/noise *ratio* was greater than five.

c The sequences were determined among the phage-ELISA positive clones. For each target, the number of distinct sequences is indicated over the total number of sequences determined.

d The soluble expression of phage-ELISA positive clones was probed using CoFi blot or Western blot experiments after liquid expression cultures. Reported *ratios* indicate the number soluble proteins over the number of clones tested.

e The properties of the clones used for further characterization were determined by ITC, DSC and/or SEC as described below. The number of internal repeats for each binder is indicated in parentheses.

For targets A3, NCS-3.24 and Ebs1, the proteins were immobilized on immunoplates and submitted to three rounds of panning with αRep libraries. In the first two rounds of panning, bound phages were eluted using an acidic glycine buffer. In the third one, bound phages were specifically eluted following incubation with an excess of free target. The binding properties of randomly picked clones from selection outputs were analyzed by phage-ELISA.

For the selection against EGFP, we developed a modified selection process in which the target was biotinylated *in vivo* and immobilized on streptavidin-coated plates. As direct coating of the protein target on the plastic wells can be problematic with unstable proteins, which may be absorbed in denatured conformations, such a selection procedure could be crucial with fragile protein targets. A target-accepting vector was constructed, in which any target sequence can easily be sub-cloned using a LIC treatment. The corresponding protein can then be expressed fused to an N-terminal biotinylation Tag (AviTag^TM^) followed by a specific peptide substrate of the TEV protease [36], [37]. This approach was developed using EGFP, as expression and immobilization of this fluorescent protein is easily visualized. The gene coding for EGFP was sub-cloned in this vector and the protein was efficiently expressed in a biotinylated form using a BirA expressing *E. coli* strain. The cells were lysed and the corresponding soluble bacterial fraction was directly incubated on a streptavidin-coated microplate without any prior purification. The selection procedure was then performed as described for other targets except for the elution step. For each round, the bound phages were specifically eluted by TEV protease cleavage of the streptavidin bound EGFP. The clones obtained after the third round of panning were analyzed by phage-ELISA.

Significant positive phage-ELISA signals were obtained for all four targets, indicating that the αRep libraries contain potential binders for each protein target. Using the optimized library (Lib2.1), three rounds were sufficient for most targets. Different ratios of positive clones were obtained ranging from 13% for EGFP to 32% for Ebs1 (Table 2). With target NCS-3.24, only Lib1.0 was used, and a unique positive clone was obtained by phage-ELISA screening after the third round of selection. This single clone was further characterized.

We also tested with target A3 whether binders could be selected from Lib1.0 and Lib2.0. The proportion of positive clones was higher with the second-generation Lib2.0 (51%) than with the initial Lib1.0 (27%) (Table 2); this suggests that using an improved diversification scheme favored a rapid enrichment of the phage population in effective binders.

### Analysis of phage-ELISA positive clones

The clones giving positive signals in phage-ELISA were sequenced. The binding properties of the proteins expressed as isolated proteins (not fused to phage particles) were screened either by ELISA for EGFP binders or by colony filtration blot (CoFi Blot) for A3 and Ebs1 binders (Table 2). For the ELISA test, the proteins were expressed following IPTG induction and the soluble bacterial extracts were incubated on target-coated immunoplates. Anti-Flag-tag or anti-His-tag antibodies were used to detect the αRep binders. The CoFi blot experiment was performed as previously described [32] but two different modes of revelation of the nitrocellulose membrane were used: an anti-His-tag antibody was employed to check that αReps were expressed as soluble proteins and specific binding clones were revealed using the biotinylated targets followed by a fluorescent streptavidin. These secondary screens revealed positive clones, all expressed as soluble proteins.

Combining the sequencing results and the first screening tests, we chose to further characterize a limited number of binders (Table 2). Binders were named using “b” for binder, the target name and a number used in the screening process. The corresponding genes were sub-cloned in a cytoplasmic expression vector (pQE-31). The proteins were over-expressed in a soluble form and purified using an IMAC followed by SEC. Binders from the two different libraries have similar expression and biophysical properties but clearly differ in terms of number of modules: binders from Lib1.0 contain 1 or 2 modules inserted between N- and C-caps, while binders selected from Lib2.1 commonly display up to 7 internal modules. Specific binding properties (affinity/specificity) of these αRep proteins were tested for their respective targets and binders were found to have affinities ranging from micromolar to low nanomolar K_d_ (Table 3).

**Table 3:** Properties of the selected binders.

Target	αRep Binder	Library^a^	Internal motifs^b^	K_d_ (nM)^c^	Complex structure^d^	Sequence of the variable positions^e^							
						Motif n°	18	19	22	23	26	30	
A3	bA3–2	Lib1.0	1	3.7±0.4	Pdb code	N-cap	S	V	K	A	V	E	
					4JW2	1	Q	F	I	A	W	K	
	bA3–17	Lib2.0	2	141±18		N-cap	W	Q	R	V	E	K	
						1	L	A	R	N	L	K	
						2	P	F	R	V	Q	K	
NCS-3.24	bNCS-16	Lib1.0	2	1450±300	Pdb code	N-cap	Y	F	R	A	A	K	
					4JW3	1	R	F	S	S	Y	E	
						2	W	F	R	A	V	E	
EGFP	bGFP-A	Lib2.1	6	15±4		N-cap	P	P	V	Y	F	K	
						1	A	S	Y	A	T	Q	
						2	G	Y	T	A	E	Q	
						3	P	W	L	T	R	E	
						4	P	W	L	T	R	Q	
						5	A	S	K	A	V	Q	
						6	E	Y	Q	R	S	K	
Ebs1	bEbs1-6	Lib2.1	7	12.3 ±12		N-cap	L	L	F	D	V	K	
						1	W	L	F	S	W	E	
						2	S	A	T	A	L	K	
						3	A	S	E	E	R	K	
						4	A	A	Y	G	A	E	
						5	R	Q	S	A	D	E	
						6	E	F	F	S	D	E	
						7	A	S	F	S	E	E	

a The library form which the binder was selected is indicated.

b The number of internal repeats inserted between the N-cap and the C-cap is reported for each binder.

c The K_d_ values were determined by ITC and result from the fitting of individual experiments. For the titration of bA3–2/A3 the value obtained in the competition experiment (Fig. 3) is reported here.

d The RX structures of two αRep/Target complexes were determined.

e For each binder, the residues found in the variable positions in the N-cap and the internal repeats are indicated in one-letter code.

### Interactions of αRep binders with their targets

#### Specific high-affinity A3 binders

**Figure 2:**
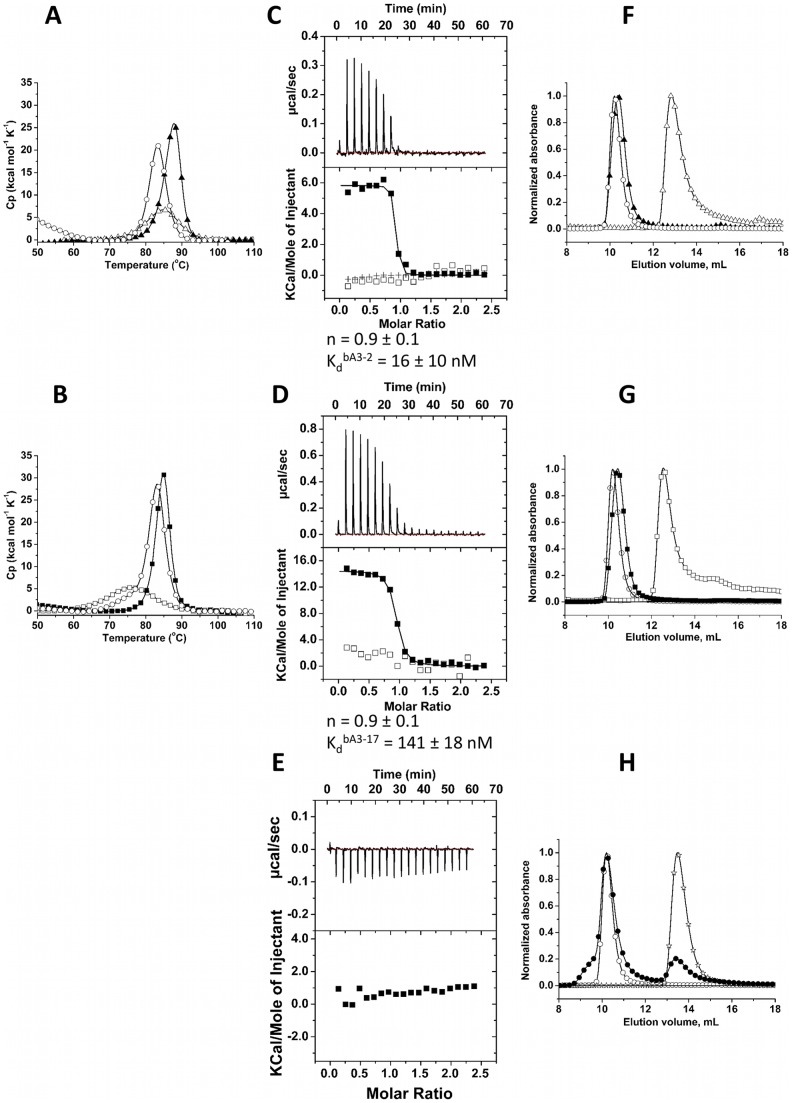
Biophysical characterization of the bA3–2, bA3–17 and A3/bA3–2 or bA3–17 complexes. (A–B) Heat denaturation of isolated proteins and complexes as assessed by microcalorimetry (DSC). (Fig. 2–A; O): A3, (0.88 mg mL-1). (Fig. 2–A; Δ): bA3–2, (2.53 mg mL-1). (Fig. 2–A; ▴): mixture A3/bA3–2 (0.88 mg mL-1/0.47 mg mL-1). (Fig. 2–B; O): A3 (0.22 mg mL-1). (Fig. 2–B; □): bA3–17, 0.15 mg mL-1. (Fig. 2–B; ▪): mixture A3/bA3–17 (0.22 mg mL-1/0.15 mg mL-1). (C-E) A3 interactions with bA3–2 and bA3–17 analyzed by microcalorimetry (ITC). For each ITC experiment, the raw data presented in the upper panel have been integrated in order to obtain the saturation curve presented in the lower panel. Parameters of each binding reaction, K_d_ and stoichiometry (n) are shown under the corresponding panel. Fig. 2–C (▪): Calorimetric titrations of binder bA3–2 (30 μM;) with A3 (350 μM). (+): experimental data corresponding to direct injection of A3 (390 μM) in the buffer. (□): binding specificity tested by titration of bA3–2 (30 μM) with NCS-wt (350 μM) as an irrelevant target. Fig. 2–D (▪): Calorimetric titrations of binder bA3–17 (30 μM) with A3 (350 μM). (□): binding specificity tested by titration of bA3–17 (30 μM) with NCS-wt (350 μM) as an irrelevant target.Fig. 2–E (▪): A3-binding specificity was evaluated by ITC analysis of the mixture of A3 (390 μM) and irrelevant substrate bNCS-16 (25 μM). (F–H) Size Exclusion Chromatography Solutions of proteins (100 μL of proteins) were injected into an analytical Superdex 75 (10/300) column equilibrated in PBS. Fig. 2–F: A3 and bA3–2. (O): elution profile of A3 alone (2.5 nmol). (Δ) elution profile of bA3–2 alone (4.5 nmol). (▴): SEC Elution profile of the ITC- mixture of A3 (2.5 nmol) and the binder bA3–2 (8.4 nmol).Fig. 2–G: A3 and bA3–17. (O): elution profile of A3 alone (2.5 nmol). (□): elution profile of bA3–17 alone (3 nmol). (▪): SEC elution profile of the ITC-mixture of A3 (2.5 nmol) and the binder bA3–17 (8.4 nmol). Fig.2–H: A3 and the irrelevant protein bNCS-16. (O): elution profile of A3 alone (2.5 nmol). (☆): elution profile of the bNCS-16 alone (4 nmol). (•): SEC elution profile of the ITC-mixture of A3 (2.5 nmol) and the irrelevant binder bNCS-16 (6 nmol).

First, the stabilities of the selected binders bA3–2 and bA3–17 and of the binder-target complexes were assessed using differential scanning calorimetry (DSC). Binders bA3–2 (Fig. 2–A) and bA3–17 (Fig. 2–B) displayed a single transition centered at 84.60±0.02°C and 75.90±0.05°C associated with an enthalpy of 80.2±0.3 kcal mol^−1^ and 85.8±0.6 kcal mol^−1^ respectively. The bA3–2/A3 and bA3–17/A3 complexes displayed a single transition centered at 87.44±0.03°C and 84.74±0.01°C, respectively. The significant shift of melting temperature (Tm) to higher temperature, together with the fact that the complexes displayed a single transition associated with an increased enthalpy (ΔHcal of bA3–2/A3 is 155±2 kcal mol^−1^) is an indication of the significant stabilizing effect of complex formation relatively to the isolated partner.

To characterize more precisely these interactions between the target A3 and its binders bA3–2 and bA3–17, the dissociation constant (K_d_) values and the stoichiometry (n) were determined using ITC. Figure 2 presents the titration of bA3–2 with the target A3 (Fig. 2–C), and the titration of bA3–17 with A3 (Fig. 2–D). The K_d_ and n values are 16±10 nM and 0.9±0.1 for A3/bA3–2 binding and 141±18 nM and 0.9±0.1 for A3/bA3–17 binding, respectively. The direct titration of A3 in the buffer showed that no significant signal due to the dissociation of A3 homodimer upon injection was observed in these ITC conditions (Fig. 2–C).

**Figure 3:**
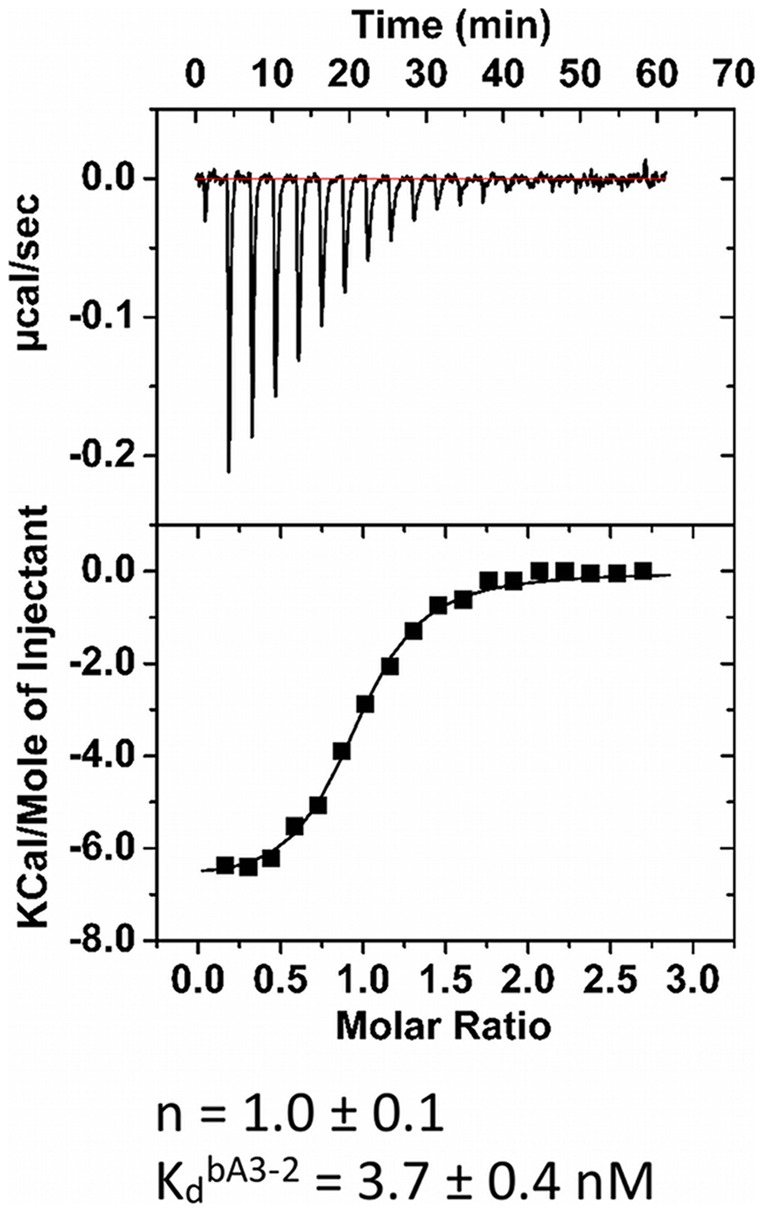
Calorimetric data for competition binding experiments. ITC titration of a mixture of A3 (25 μM) pre-bound to bA3–17 (43 μM) with bA3–2 (350 μM). In these conditions, the apparent binding constant for bA3–2 decreases within the range required for ITC. Determination of the K_app_ is given by: K_app_  = K_a_^bA3–2^/(1+ K_a_^bA3–17^ [bA3–17]). ΔH^bA3–17^ and K_a_^bA3–17^ used in the data analysis had been determined in Fig. 2–B.

Two types of ITC control experiments were performed to confirm the affinity/specificity of the selected binders bA3–2 and bA3–17 to A3. First, bA3–2 and bA3–17 were titrated with NCS-wt used as a non-relevant target (Fig. 2–C; Fig. 2–D). Second, αRep protein (bNCS-16) selected to bind a protein unrelated to A3 was titrated with A3 (Fig. 2–E). Results showed, as expected, that no interactions occurred in both conditions. For the A3/bA3–2 transition, due to the high affinity of this complex, too few points were collected near equivalence to precisely evaluate the dissociation constant K_d_. To circumvent this problem, the binding thermodynamics of the high affinity ligand bA3–2 was measured using a competition experiment with the A3 protein pre-bound to the weaker binder bA3–17. The titration of the bA3–17/A3 mixture with bA3–2 is presented in Figure 3. The heat-binding isotherm of this titration has an optimal curvature to allow the determination of the apparent tight binding affinity (K_d_ = 3.7±0.4 nM) of bA3–2 to A3. The stoichiometry observed for the A3/bA3–2 (Fig. 2–C) and A3/bA3–17 complexes (Fig. 2–D) corresponds to one monomer of target for one monomer of binder. This suggested the formation of heterodimers composed of a monomer of A3 and a monomer of binder.

Analytical SEC was used to compare the elution volumes of the complexes bA3–2/A3 (Fig. 2–F) and bA3–17/A3 (Fig. 2–G) to each protein alone. Previous results [32] had shown that A3 protein is dimeric in low micromolar concentrations. Thus the elution volume of this protein corresponded to a dimer of A3. The samples obtained after ITC titrations were analyzed in analytical SEC experiment. In this sample, the peak corresponding to the binder alone disappeared, whereas a new peak was eluted at a lower volume, *i.e*. with a higher Stokes radius. This peak was also slightly shifted to a higher volume compared to the A3 peak (Fig. 2–G). This is consistent with the formation of heterodimers composed of one copy of A3 monomer plus one copy of binder bA3–2 or bA3–17/A3. As a control, no interaction is observed between A3 and bNCS-16, as the mixture presented two separate peaks corresponding to the elution volume of each protein alone (Fig. 2–H).

#### A variant-specific NCS binder

**Figure 4:**
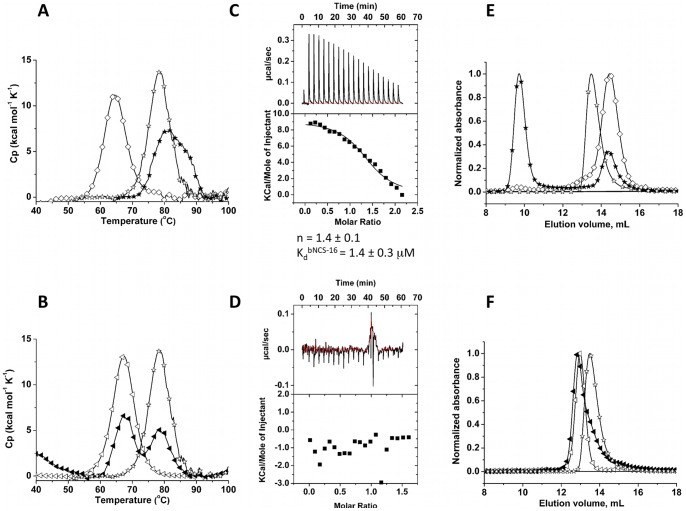
Biophysical characterization of the bNCS-16 and NCS-3.24/bNCS-16 complex. Fig. 4–A: Heat denaturation of binder/target (bNCS-16/NCS-3.24) pair assessed by DSC. (**⋄**): NCS-3.24 (0.13 mg mL^−1^). (☆): selected bNCS-16, 0.15 mg mL^−1^. (★) mixture NCS-3.24/bNCS-16 (0.13 mg mL^−1^/.0.15 mg mL^−1^). Fig. 4–B: Heat denaturation of bNCS-16/NCS-wt assessed by DSC. (**◃**): NCS-wt. (☆): bNCS-16 (0.15 mg mL^−1^). (◂) mixture NCS-wt/bNCS-16 (0.13 mg mL^−1^/0.15 mg mL^−1^). Fig. 4–C: ITC calorimetric titrations of binder bNCS-16 (20 μM) with NCS-3.24 (211 μM). Fig. 4–D: The NCS-3.24-binding specificity was evaluated by ITC analysis of injection of NCS-wt (350 μM) in a solution of bNCS-16 (44.5 μM). (E–F) Size Exclusion Chromatography (Superdex 75 10/300.) of the selected bNCS-16 with NCS-3.24 (E) or NCS-wt (F). Fig. 4–E. (☆): elution profile of the bNCS-16 alone (4 nmol). (**⋄**): elution profile of the NCS-3.24 alone (1.25 nmol). (★): SEC Elution profile of the ITC- mixture of NCS-3.24 (8.4 nmol) and the binder bNCS-16 (4.8 nmol). Fig. 4–F. (**◃**): elution profile of the NCS-wt alone (5 nmol). (☆): elution profile of the bNCS-16 alone (4 nmol). (◂): SEC elution profile of the ITC-mixture of NCS-wt (14 nmol) and the binder bNCS-16 (10.7 nmol).

In a DSC experiment, the binder bNCS-16 and the target NCS-3.24 displayed a single transition centered at 78.41±0.02°C and 64.72±0.02°C associated with an enthalpy of 121±6 kcal mol^−1^ and 99.9±5.6 kcal mol^−1^ (Fig. 4–A) respectively. The mixture bNCS-16/NCS-3.24 presented a single transition centered at 82.08±0.09°C associated with a ΔHcal of 92.8±1.5 kcal mol^−1^. The shift of the mixture thermogram is consistent with the formation of a stabilized complex between both proteins. As a control, the same experiments were performed using NCS-wt instead of NCS-3.24 (Fig. 4–B). Two separated peaks were observed in the mixture, corresponding to thermograms of each protein done separately. The stabilization effect is therefore specifically related to the presence of a complex of αRep with its cognate target NCS-3.24 and is not observed in the absence of interactions with the related protein NCS-wt.

ITC titration of bNCS-16 with NCS-3.24 (Fig. 4–C) yielded K_d_ and n values of 1.4±0.3 μM and 1.4±0.1 respectively. To investigate target specificity of bNCS-16, titration was performed with NCS-wt (Fig. 4–D), which had similar overall structure but differs from NCS-3.24 by 7 residues, all located in the same crevice on NCS surface. No interaction occurred between NCS-wt and bNCS-16 suggesting that αRep binders can discriminate between closely related proteins. This also indicates that αRep proteins are able to specifically recognize, not only convex surfaces as expected, but also concave surfaces, where the substituted side chains are located.

A solution containing bNCS-16 with NCS-3.24 was injected on an analytical SEC column. In the SEC curve, no peak corresponding to the binder alone was observed (Fig. 4–E). A new peak eluted at a lower volume compared to those of the target or binder protein alone. This is consistent with the formation of a complex. No similar complex peak was observed when NCS-wt was injected instead of NCS-3.24 (Fig. 4–F).

#### A GFP binder

**Figure 5:**
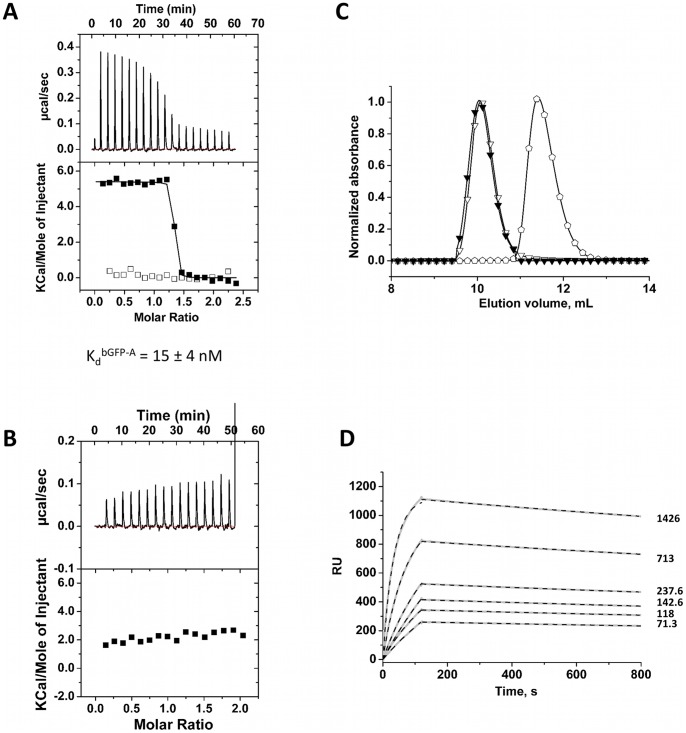
Biophysical characterization of the bGFP-A and GFP/bGFP-A complex. (A) ITC calorimetric titrations. Concentrations values are expressed in monomer concentrations. (▪): Tiration of GFP (35 μM) with bGFP-A (350 μM). (□): The bGFP-A binding specificity was tested by titration with NCS-wt (bGFP-A 30 μM, NCS-wt 350 μM). (B) The GFP-binding specificity was evaluated by ITC analysis of injection of bA3–1 (360 μM) in a solution of GFP (30 μM). (C) Size Exclusion Chromatography (Superdex 75 10/300) of the selected bGFP-A and GFP. (▾): SEC Elution profile of a mixture of GFP (2.25 nmol) and the binder bGFP-A (6.75 nmol). (∇): elution profile of the bGFP-A alone (2.25 nmol). (**
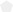
**): elution profile of the GFP alone (6.75 nmol). (D) Affinity determination of selected bGFP-A using SPR. Different concentrations of bGFP-A (71,3; 118; 142,6; 237,6; 713; 1426 nM) were applied to flow cell with immobilized biotinylated EGFP for 120 s followed by washing buffer flow. The sensorgrams were corrected for non-specific binding by subtraction of a channel without EGFP bound (grey curve). The fits of k_on_ and k_off_ rates are indicated by black dashed line. K_d_ values were computed using k_off_  = 1.7×10^−4^ s^−1^ for all concentrations and k_on_  = 4.3, 4, 2.2, 2.6, 2, 2×10^4^M^−1^ s^−1^ for the increasing concentrations respectively.

The aim of this selection was to isolate specific binders of GFP. Binder bGFP-A was selected as described above. ITC titration for EGFP with bGFP-A displayed a K_d_ of 15±4 nM and n values of 1.1±0.1 (Fig. 5–A). The binding specificity of the binder bGFP-A was tested, using NCS-wt as a titrant (Fig. 5–A). No interaction was observed in these conditions. An ITC control experiment done with an αRep not selected against EGFP showed, as expected, no ITC signals (Fig. 5–B).

Analytical SEC was used to compare the elution volumes of the complexes bGFP-A/EGFP (Fig. 5–C) to each protein alone. The molecular mass of bGFP-A is similar to EGFP, respectively 25,6 and 27.9 kDa; however bGFP-A was eluted in a lower volume compared to EGFP. This suggested that this protein is homodimeric. SEC analysis of an EGFP/bGFP-A sample showed that the peak corresponding to EGFP disappeared, whereas a new peak was eluted in a lower volume superimposed to the bGFP-A binder. This is consistent with the formation of a target/binder complex as a heterodimer composed of one copy of bGFP-A monomer plus one copy of the GFP protein. Therefore this suggests that bGFP-A is a homodimeric protein and has to dissociate in order to make a 1/1 complex with its target.

The binder bGFP-A shows a high affinity for EGFP, at the limit of the ITC detection. Surface Plasmon Resonance (SPR) experiment of this binder/target pair was carried out to support the ITC results (Fig. 5–D). Analysis was performed on a sensor surface with covalently bound streptavidin to capture biotinylated EGFP. bGFP-A was then flowed across the chip.

The binder showed specific and reversible binding to its target. The rate constants were k_on_ = 3±1×10^4^ M^−1^ s^−1^ for association, and k_off_ = 1.7±0.1±10^−4^ s^−1^ for dissociation, corresponding to an equilibrium dissociation constant K_d_ of 6±2 nM.

#### Ebs1 binders

**Figure 6:**
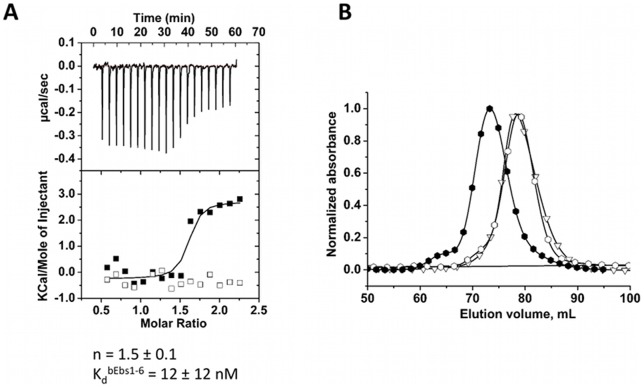
Biophysical characterization of the bEbs1-6 and Ebs1/bEbs1–6 complex. (A) ITC calorimetric titrations. (▪): Titration of Ebs1 (35 μM) with bEbs1–6 (387 μM). (□): The bEbs1–6 (30 μM) binding specificity was tested by titration with NCS-wt (350 μM). (B) Size Exclusion Chromatography (Superdex 200 prep grade Hiload 16/60) of the selected bEbs1–6 and Ebs1 (?):SEC elution profile of bEbs1–6 alone; (∇): elution profile of Ebs1 alone; (?): elution profile of an equimolar mixture of Ebs1 (55 nmol) and the binder bEbs1–6 (55 nmol).

The binder bEbs1–6 selected using Ebs1 as target was characterized by ITC (Fig. 6–A). Binder bEbs1–6 displayed a K_d_ and n values 12±12 nM and 1.5±0.1 respectively. The binding specificity of the bEbs1–6 was also tested, using a titration with the non-relevant target NCS-wt (Fig. 6–A). No interaction was observed in these conditions.

Interactions were also controlled by SEC. The injection of an equimolar Ebs1:bEbs1–6 mixture resulted in a new peak, eluted in a lower volume compared to each protein alone (Fig. 6–B). This is consistent with the formation of a heterodimer. Interaction was additionally monitored using SEC-MALLS (data not shown). The molecular mass of Ebs1 determined by SEC-MALLS was 62 kDa, close to the calculated theoretical mass (70 kDa). The molecular mass of the binder bEbs1–6 determined by SEC-MALLS is 54 kDa corresponding to a homodimer (calculated mass for a monomer is 31 kDa). Analysis of the binder/target sample revealed a major peak centered at 107 kDa, consistent with a heterodimeric complex composed of one copy of target monomer and one copy of binder monomer (calculated mass of a 1∶1 complex is 101 kDa).

### X-ray structure of αRep/target complexes

#### A3/bA3–2 complex

**Figure 7:**
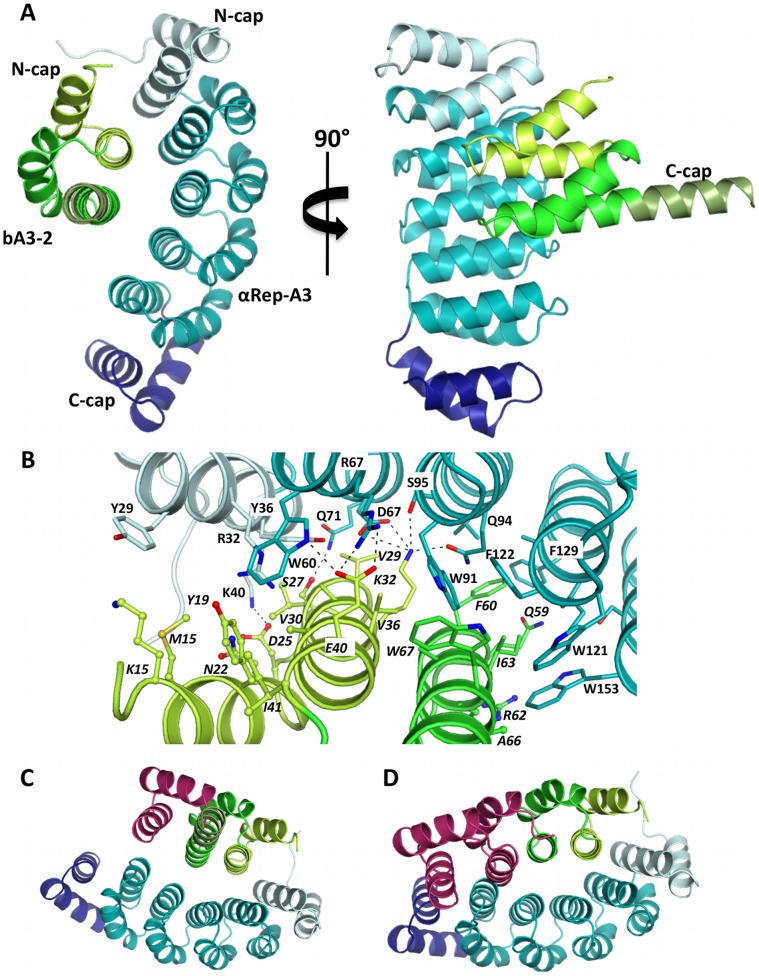
Representation of the bA3–2/A3 complex. (A) Ribbon representation of the bA3–2/A3 complex. A3 is represented in light blue (Ncap in grey and C-cap in deep blue). bA3–2 is in green. (B) Representation of the interface between bA3–2 and A3 proteins (same colour code as panel A). Residues involved in the interaction are shown as sticks. Residues from the interface, which belong to the invariant scaffold of αRep proteins, are shown as ball and sticks. Hydrogen bonds are depicted by dashed lines. (C) Modelisation of a canonical bA3–2 C-cap (magenta) in the structural context of the complex. (D) Modelisation of an additional HEAT repeat module and of a C-cap (magenta) in the structure of the αRep protein bound to A3.

The crystal structure of the A3/bA3–2 complex was solved to 1.9 Å resolution with one copy of the heterodimeric complex in the asymmetric unit. The A3 structure in complex with bA3–2 is very similar to the previously solved structure of the uncomplexed form (rmsd of 0.6 Å over 186 Cα atoms [32]). The main discrepancy between the complexed and uncomplexed form of A3 is found at the N-terminal extremity (residues 7 to 10), which corresponds to part of the hexahistidine tag. The bA3–2 protein belongs to the same structural family as A3 but contains only one module between its N- and C-caps. Surprisingly, the first helix of the C-cap of bA3–2 (residues 73 to 85) flips by 180° relative to its location in the C-cap of A3 and forms an extension of the second helix from the preceding repeat (Fig. 7–A). The second α-helix (residues 86 to 108) from the bA3–2 C-cap has no defined electron density and mass spectrometry analysis revealed that the region encompassing residues 94 to 108 was proteolytically removed in this protein sample, presumably during crystallization. Despite this difference, bA3–2 is structurally very similar to A3 both in its apo-form (rmsd of 0.3 Å over 60 Cα atoms) or bound to bA3–2 (rmsd of 0.5 Å over 60 Cα atoms). bA3–2 and A3 interact *via* their concave faces which encompass the surface residues that are allowed to evolve (Fig. 7–A). The complex has an interface area of 860 Å^2^ (corresponding to a total surface area excluded from the solvent upon complex formation of 1720 Å^2^), which is typical of protein-protein complexes [38]. Complex formation involves 19 residues located between the N-cap and the 4^th^ repeat for A3 and 22 residues located within the N-cap and the repeat from bA3–2 (Fig. 7–B). Of the 19 residues from A3 involved in the interface, four originate from the N-cap and 15 from the internal repeats. Interestingly, all are found at randomized positions from A3. As for bA3–2, half of the residues involved in A3 binding (11 out of 22) are from randomized positions and two-third (15 out of 22) are located within the N-cap, which plays a predominant role in A3 binding. The interface is mainly hydrophobic (70% of interface atoms being non-polar atoms) and involves the following hydrophobic residues Tyr29, Tyr36, Trp60, Trp91, Val98, Trp121, Phe122, Ile125, Phe129 and Trp153 from A3 and Met15 Tyr19, Val29, Val30, Val36, Phe60, Ile63 and Trp67 from bA3–2 (Fig.7–B). However, four hydrogen bonds and three salt-bridges are also involved. Modelling of the C-cap structure from bA3–2 based on the structure of the corresponding region from A3 does not reveal any steric clash between this “modelled” bA3–2 C-cap and the A3 protein. This clearly indicates that degradation of part of the C-cap, as well as unfolding of the remaining part, is not required for complex formation (Fig. 7–C), and that a non-cleaved “canonical” C-cap is compatible with binding to bA3–2, as expected from solution studies. Conversely, the replacement of bA3–2 by models of αRep proteins composed of two or more internal repeats (as would be the case for the other A3 binder bA3–17 identified in this study) is not possible in this arrangement. Docking of αRep constructs that are longer than bA3–2 onto the A3 protein in the complex indicates that the presence of an additional internal repeat would introduce steric clashes with the fourth repeat of A3 (Fig. 7–D). Hence, we conclude that the bA3–17 protein will bind A3 in a completely different manner than bA3–2.

#### NCS-3.24/bNCS-16 complex

**Figure 8:**
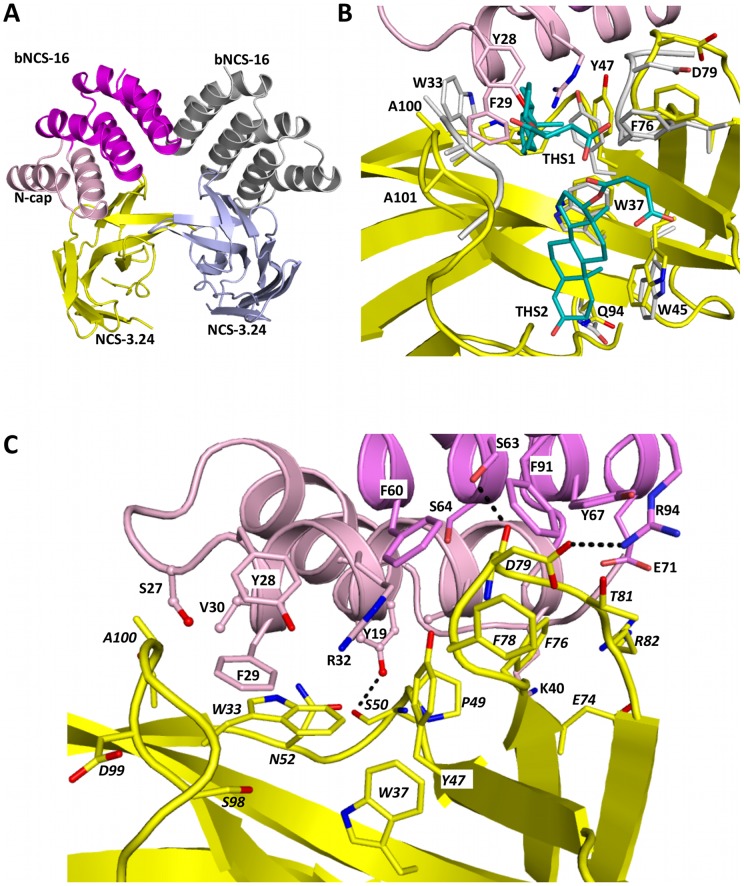
Representation of the bNCS-16/NCS-3.24 complex. (A) Ribbon representation of the two bNCS-16/NCS-3.24 complexes present in the asymmetric unit. (B) Comparison of the NCS-3.24/testosterone complex (NCS-3.24 is in grey and testosterone hemisuccinate are in blue) with the bNCS-16/NCS-3.24 complex (same colour code as panel A). For clarity, only loops from NCS-3.24 that undergo conformational changes are shown.(C) Representation of the interface between bNCS-16 and NCS-3.24 (same colour code as panel A). Residues involved in the interaction are shown as sticks. Residues from the interface, which belong to the invariant scaffold of αRep proteins are shown as ball and sticks. Hydrogen bonds are depicted by dashed lines.

The structure of the NCS-3.24/bNCS-16 complex was determined to 2.6 Å resolution with two copies of the heterodimer in the asymmetric unit (Fig. 8–A). The two complexes are related by a two-fold axis and are virtually identical (rmsd of 0.3 Å over 200 Cα atoms). Comparison of the structure of the NCS-3.24 protein in this complex to the previously solved crystal structures of this NCS variant shows that this protein does not undergo drastic structural rearrangements (rmsd of 0.7–0.8 Å over 110 Cα atoms (Fig. 8–B, [39]) except for the loops encompassing residues Ala75-Asp79 and Ala100-Ala101, which are involved in testosterone binding. These two loops move so as to slightly open the steroid-binding crevice in the NCS-3.24/bNCS-16 complex. The structure of bNCS-16 is very similar to those of A3 and bA3-2 (rmsd of 0.4–0.7 Å over 60–95 Cα atoms). In the structure of the NCS-3.24/bNCS-16 complex, the C-cap of the αRep protein (residues to 105 to 139) is not visible in the electron density due to partial degradation (residues 125 to 139, as observed for bA3-2) and to high flexibility of the remaining region (residues 105 to 124). Modelling shows that the presence of a structured C-cap would not preclude formation of the observed heterodimer (data not shown) but would prevent formation of the hetero-tetramer observed in the asymmetric unit (see below). Hence, the unfolding of the C-cap region encompassing residues 105–124 probably results from the partial hydrolysis of the C-cap during crystallization and the partially truncated C-cap form favours hetero-tetramers association compatible with packing in this crystalline form. Beyond the predominant NCS-3.24/bNCS-16 interface, the largest interface area is found between the two NCS-3.24 molecules (565 Å^2^).

The NCS-3.24/bNCS-16 complex involves an interface area of 720 Å^2^, with 17 residues from NCS-3.24 contacting 17 residues from bNCS-16 (Fig. 8–C). The interface is mainly hydrophobic (Trp33, Trp37, Tyr47, Pro49, Phe76, Phe78 from NCS-3.24 and Tyr19, Tyr28, Val30, Phe60, Tyr67, Phe91 from bNCS-16). Two hydrogen bonds (between carbonyl atoms from Ala50 and Asp79 from NCS-3.24 and Tyr19 and Ser63 hydroxyl groups from bNCS-16, respectively) and one salt bridge (between Asp79 from NCS-3.24 and Arg94 from bNCS-16) are also involved. Ten out of 17 interacting residues from bNCS-16 locate within the N-cap of the protein while 5 and 2 interacting residues are from internal αRep repeats 1 and 2, respectively. Thirteen of these residues are found at variable positions.

We then compared the present NCS-3.24/bNCS-16 complex with the previously solved structure of NCS-3.24 bound to two testosterone hemisuccinate (THS1 and THS2) molecules or to a MES buffer molecule [39] with the aim of deciphering the specificity of the bNCS-16 protein for NCS-3.24 variant versus NCS-wt. Among the eighteen residues from NCS-3.24 involved in bNCS-16 binding, six correspond to “variable” NCS positions (Trp33, Trp37, Tyr47, Asn52, Phe78 and Ser98), and are therefore different from the homologous positions in the wild-type NCS, explaining why NCS-wt is not recognized by bNCS-16. Superimposition of the NCS-3.24/THS complex onto the NCS-3.24/bNCS-16 complex reveals that the THS1 ligand binding cleft is slightly more open in the NCS-3.24/bNCS-16 complex than in the NCS-3.24/THS complex (Fig. 8–B). Furthermore, NCS-3.24 in complex with bNCS-16 would probably not bind THS1 because several steric clashes would be created with the second helix of the N-cap (in particular Tyr28 and Phe29 side chains; Fig. 8–B). In addition, the side chain of Trp33 from NCS-3.24, which runs parallel to the THS1 steroid rings and hence plays a predominant role in its binding [39] rearranges by 90° so as to interact with helix 2 from the N-cap and is no longer available for the stabilization of THS1 (Fig. 8–B). The same is not true for the THS2 binding site. Indeed, this second site is remote from the interface with the bNCS-16 protein and the side chains from NCS-3.24 residues involved in the interaction with THS2 adopt exactly the same conformation (Fig. 8–B).

## Discussion

### αRep as a versatile scaffold

αRep proteins were previously described to possess a stable fold, compatible with an extended variability both in repeat sequence and repeat number [32]. Here, we demonstrate that it is possible to select αRep proteins binding tightly and specifically to four different, non-related and arbitrarily chosen protein targets. Binders selected by three rounds of phage display, without affinity maturation steps, bind their cognate target proteins with affinities corresponding to K_d_ values comprised from micromolar (bNCS-16, 1.45 μM) to nanomolar (bA3−17, 141 nM; bGFP-A, 15 nM; bEbs1–6, 12 nM; bA3–2, 3.7 nM) ranges.

Coiled coils or inter-helical assemblies are commonly found in natural protein complexes and, therefore, one might suspect that αRep proteins could bind only to alpha-helicoidal protein targets. However, the present results show that binders were obtained for targets with different topologies: an all-α repeat protein (αRep-A3), a β-sandwich (NCS-3.24), a β-barrel (EGFP) and a large all-α protein with an unknown structure (Ebs1). Therefore, as previously observed with affibodies [14], [40] the surface on the protein target eligible to give rise to contact with αRep surfaces (the “αRepotope”) does not have to be helical. Moreover, this further supports the view that, although mammal's antibodies are based on IG domains, general protein recognition is not the privilege of this type of architecture. The presence of a hypervariable sequence supported on loops or on β-strands is not mandatory to make an efficient binding surface and a versatile protein scaffold.

### Libraries and selections

Selections were first performed with the previously described library (Lib1.0) using αRep-A3 and NCS-3.24 as targets. Several A3 binders and a single NCS-3.24 binder were obtained suggesting that the αRep scaffold could be an efficient source of specific proteins. These results motivated the construction of a larger and improved library useful as a generic source of potential binders.

This optimized library (Lib2.1) is more diverse and comprises proteins with more than 3 repeats, which were less frequent in the initial libraries Lib1.0 and Lib2.0. The procedures used to shuffle the subset of correct sequences of Lib2.0 and recombine only the useful part of the initial diversity is potentially applicable to other libraries, including non-repeated folds, provided that suitable restriction sites are included in the library design.

The propensity of long repeat proteins to generate binders has not yet been experimentally explored. All previously reported repeat protein libraries [30], [31], [41]–[43] were designed with a constant number of repeats while αRep Lib2.1 includes protein with variable number of repeats. The extended binding surfaces available on longer proteins may provide the structural basis for a high binding energy and it seems therefore attractive to extend the binding surface. However, the experimental sampling of the sequence space of proteins with many repeats is very sparse. It is therefore highly unlikely that any selected long protein could have the optimal combination for all of its variable side chains. Before doing selection experiments with Lib2.1, it was impossible to predict on reliable theoretical grounds how the tradeoff between surface extension and sparse sampling of long proteins would practically impact selection outcome.

The experimental results show that for each target, binders were obtained with a size distribution varying from one to seven variable internal repeats. In addition to the four targets described, selection rounds were recently performed with the optimized library Lib2.1 against a set of six other targets and positive clones in phage-ELISA experiments were found in all cases (data not shown). Further characterization of the corresponding binders is currently in progress. But it is already clear that with Lib2.1 rather long proteins are commonly, albeit non-exclusively, selected. Therefore, although the corresponding sequence space is very sparsely sampled, the fraction of long proteins present in Lib2.1 is an efficient source of binders.

For some applications requiring a high fraction of binding capacity per mass unit, the selection of large binders may actually be viewed as a drawback of this type of libraries. For other applications, like crystallization chaperones, where the extent of target surface covered by large binders could strongly modulate intermolecular contact, this type of library may be particularly interesting. Are all the αRep motifs of long binders involved in target interactions? The question remains to be further investigated by a detailed characterization of hot spots in these extended binding surfaces. As supported by Grove *et al.* [44], natural repeat proteins with more repeated motifs forming an extended surface can generate an efficient route to tight binding with a variety of ligands. For example, karyopherin β2 is a protein organized with 20 consecutive HEAT repeats involved in multiple ligand complexes with nucleoporin, NLS (nuclear localization signals) of cargo proteins and the regulatory protein Ran-GTP [44], [45]. The protein is divided in 3 major segments along the solenoid axis of the structure (repeat 1–8, 9–13 and 14–18) to achieve these functions [45]. Another example is the protein phosphatase 2A (PP2A), a heterotrimeric protein, in which the A subunit (with 15 HEAT repeats) binds to both the regulatory ‘B’ subunit and the catalytic ‘C’ subunit with different sets of HEAT repeats [46]. The αRep library Lib2.1 could provide extended platforms suitable to bind and/or stabilize protein complexes.

The recognition between each αRep and its target is dependent upon the conformational states of the target protein. Therefore, the quality of the prepared target appears crucial to get efficient binders. The stability of the target is also an important factor leading to the success of the selection. The selection approach using direct coating of the protein target was successful as binders for A3, NCS and Ebs1 were conformational binders. However, two other targets with low stabilities were recently used for selections. For these two targets, some phage-exposed proteins were found to bind to immobilized protein targets while the corresponding free proteins did not bind their targets (data not shown). These data suggest that these unstable targets were coated in non-native conformation(s) and non-native state specific binders were thus generated. As unstable proteins can be destabilized upon direct plastic coating, we set up a selection process of *in vivo* biotinylated proteins [37], [47]. This process could also be applied to stable proteins to benefit of specific elution by TEV protease cleavage of the streptavidin bound target. This approach was validated with the selection of EGFP binders without prior purification of the target. This approach, based on previously reported selection procedures [37] using peptide phage display libraries is potentially applicable to any protein that could be expressed in its folded form in *E. coli*.

### Binders properties

The selected αRep proteins present the expected valuable properties of the designed αRep proteins. They are expressed in high amounts (50–100 mg L^−1^), in a soluble and stable form (Tm >70°C; Fig. 2–A, b; Fig. 4–A). As previously observed for proteins randomly isolated from the libraries, the selected binders are monomeric (bA3–2 (Fig. 2–F); bA3–17 (Fig. 2–G); bNCS-16 (Fig. 4–E) or dimeric (bGFP-A (Fig. 5–C); bEbs1–6 (MALLS, data not shown)). All the selected proteins specifically bind to the target protein against which they were selected and do not cross-react with other proteins as shown by control experiments (Fig. 2–E, H; Fig. 4–B, D, F; Fig. 5–B).

The A3 structure was previously described as a dimer in which the subunits interface results from N-cap, C-cap and randomized side chains residues [32]. ITC and SEC experiments showed that interaction between A3 and bA3–2 (Fig. 2–F) or bA3–17 (Fig. 2–G) resulted in the formation of 1∶1 heterodimers. This suggests that the measured apparent K_d_ obtained by ITC was the result of two combined events, dissociation of the A3 homodimer followed by the tight association of each A3 monomer with bA3–2 (or bA3–17). Similarly, SEC (Fig. 5–C; Fig. 6–B) or MALLS experiments (data not shown), suggest that some selected binders (bGFP-A or bEbs1–6) are homodimers in the uncomplexed form while analysis of the interaction with their target revealed a peak consistent with a heterodimeric complex. Therefore, the K_d_ values for these interactions are presumably composite and reflect homodimer dissociation combined with target/binder heterodimer association event. Since we are able to estimate a K_d_ value of 141 nM for bA3–17, the homodimer dissociation constant of A3 is probably not lower than micromolar. Therefore, the absolute K_d_ values for bA3–2 and bA3–17 are lower but not very different from the apparent measured K_d_. These results also showed that homo-dimeric αRep proteins with relatively weak association are not rare in the libraries and can be selected as a source of high affinity monomeric binders.

EGFP and Ebs1 binders selected from Lib2.1 contain respectively 6 and 7 repeats and have affinities in the low nanomolar range while the A3 and NCS-3.24 binders selected from Lib1.0 and Lib2.0 contain 1 or 2 repeats and have affinities ranging from the micromolar to the nanomolar range. Long αRep binders presenting a large binding surface can potentially support more interactions and thus give rise to a lower K_d_ value than small αRep binders. However, the binding affinity is not systematically correlated in such a simple way to the protein size. For example, among the A3 binders, the binder with the higher affinity is the smaller one (bA3–2 vs bA317). As noted above, due to sparse sampling of a large sequence space, it is highly unlikely the optimal combination of all variable positions could be directly selected, before any additional affinity maturation steps. However this work indicates that large interface, eventually with suboptimal side chain combinations are quite commonly found as a primary hit. Such large interfaces could consequently be ideal starting points for affinity maturation processes.

The affinity of bGFP-A (K_d_ = 5.5 nM) obtained by SPR experiment is in the same range of magnitude of that found by ITC (15.4 nM). SPR offers complementary results compared to ITC, providing access to the kinetic constants of the binder/target interaction. Here, the high affinity is mostly correlated to low dissociation rate (1.7×10^−4^ s^−1^). To conclude, the affinity of bGFP-A for EGFP, obtained in the nanomolar range, shows that the modified procedure used for this selection gives rise to tight and specific binders and furthermore conveniently limits target pre-purification steps.

Finally, although no specific step was included in the selection procedure, the binder selected against the variant NCS-3.24 clearly discriminates between the target NCS-3.24 and NCS-wt. This fine-tuning of the binding interface is illustrated by the structure of the complex, showing that six residues of the interface originate from mutations in the variant design. This suggests that the αRep libraries could be a valuable source of highly specific binders for dedicated targets.

### Structure of αRep complexes

The structures of the two binder/target complexes confirm that the variegated surface of αRep is, as expected, an essential component of the binding interaction. It should be noted that, although the sequences of the N-cap and that of internal modules are distinct, their structures are very similar and includes variable positions. In both complexes, the variable N-cap positions are directly involved in the interface.

The C-cap sequence was not engineered but directly taken from the sequence of protein MtH187 used as a starting point for αRep design. In the two crystallized complexes the C-cap was cleaved and in one complex the remaining part rearranged by domain swapping to establish intermolecular contacts. We also observed in other selected αReps that, the C-terminal part is sensitive to proteolysis and may be partially cleaved during purification or crystallization, although this is highly variable between different αRep proteins. It is unclear whether this more dynamic behavior of this part of the otherwise extremely stable molecule results from the Mth187 C-cap biological function or if it is related to structural incompatibility between the C-cap and the variable residues on the penultimate repeat. Although sequences of designed ankyrins are not related to those of αReps, structural studies of designed ankyrin repeat complexes also showed that their C-caps are sometimes unfolded or cleaved [24]. In both cases, this proteolytic susceptibility may seem surprising as these proteins are extremely stable. Experimental folding studies on TPR, LRR and ANK repeats [48]–[52] have suggested that stability increases with the number of repeats, as generally observed for αRep repeats. However this overall trend can be modulated by the combination of variable positions in the sequence of each repeat. This could explain for example why some selected binders with low number of repeats (such as bA3–2–1 internal repeat -Tm  = 84.60±0.02°C obtained by DSC experiment) present higher stability than binders with more internal repeats (bA3–17–2 internal repeats – Tm  = 75.90±0.05°C).

Folding/unfolding of these proteins could be described using an Ising-like model, in which each repeat is stabilized by its neighbors. In this view, N- and C-caps located at each end of the fold are the most dynamic parts of the protein and therefore could preferentially be cleaved during a transient unfolding event. The Darpins C-cap was specifically stabilized in a second generation of these designed proteins [53].

## Conclusion

The αRep fold and the construction of an optimized αRep library provide a generic resource to select specific binders for various protein targets of unrelated structures. The selected αRep proteins bind their targets specifically with affinities from the micromolar up to the nanomolar range before any affinity maturation steps. αReps are disulfide-free, stable, highly expressed in a soluble form and are not prone to aggregation. Structures of αRep/target complexes show that conformation-dependent target recognition emerges, as expected, from the juxtaposition of the variable surface, while the structures of repeated modules remains remarkably constant. Libraries with a variable number of modules are an efficient source of binders. These binding studies together with structures of binary complex of αRep provide clear evidences that this artificial protein family is a general and versatile tool for biological recognition.

## Materials and Methods

### Library design

A collection of natural sequences was identified by a Blast search on Uniref 90 using as a probe a virtual sequence made by ten consecutive αRep motifs, with non-specified residues in variable positions. 200 sequences were collected, manually split in modules and curated from all repeat having more than three indels, leaving a collection of aligned 1719 repeats. The distribution of aminoacid in this sequence collection was computed and used as a target distribution to define the randomization scheme (Fig. 1). Diversity was encoded, position-by-position to match the target distribution. In order to limit the number of oligonucleotides used to randomize 18–19 and 22–23 positions pairs, oligonucleotides corresponding to these positions were designed by combining a set of partially randomized codons (18: hcc, kac, bcg, gwa, sgt, ama, ncg, tgg, ctg, tac, ccg; 19: gma, kcg, cwg, amc, twc, cgc, gac, tac, ggt, tgg; 22: cgt, raa, kct, cwg, tac, ttc, tgg, atc, ayg, gtt; 23: ryt, skt, gma, rmc, sag, mgc, wct, gcg, ggt, tcg, cgt, kct, gct, gaa, aac). In each case, residues with low frequency were eliminated from the encoded diversity. Aromatic side chains (Tyr and Trp) are enriched in different types of protein interfaces and therefore their proportion was increased relatively to their proportion in the natural repeats collection.

### Library construction

The construction of Lib1.0 was described previously [32]. Lib2.0 was built using the same approach except for the matrix circles used in the RCA amplification. Fully double-strand DNA circles were assembled by hybridization of degenerated primers. For the construction of Lib2.1, a solution of phages produced from Lib2.0 (4×10^13^ phages) was incubated on immunotubes coated with an anti-Flag tag antibody (10 μg mL^−1^) and blocked with a solution of TBS (20 mM Tris-HCl pH 8.0, 150 mM NaCl) containing BSA (4%) and Tween-20 (0.1%) (TBST-BSA). Proteins were exposed on the phages via a C-terminal fusion with the M13-PIII. Retained phages displayed an N-terminal Flag-Tag and thus contained a fully coding sequence. The immunotubes were washed and bound phages were eluted by an acidic glycine solution (0.1 M, pH 2.5). Freshly prepared bacteria were infected by the recovered phages and plated. Plasmids were recovered as a pool from this “filtrated” bacteria population. The αRep modules from this plasmid pool obtained by BsmBI restriction were extracted from agarose gel, circularized by self-ligation and re-amplified using RCA. This population of DNA fragments was ligated into BsmBI digested plasmids from Lib2.0. The ligated products were transformed into XL1-Blue MRF' electro-competent cells, plated on large 2YT plates supplemented with ampicillin and tetracyclin. Colonies were harvested from the plates pooled and cell suspension stored as glycerol stocks of the library Lib2.1.

### Phage display selection on purified targets

For each target, the libraries were panned using microtiter plates coated overnight at 4°C with 100 μL of the target (20 μg mL^−1^) per well. Phages from each library were prepared using XL1-Blue MRF' bacteria transformed with the phagemid libraries and infected with the helper phage Phaberge [54]. Phages were allowed to replicate overnight at 30°C. The cultures were centrifuged at 5.000 g for 30 min and the supernatants were recovered and dialyzed against TBS using a 300 kDa cut-off dialysis membrane to eliminate free proteins from the phage solution. The dialyzed phages (1 to 2×10^10^ particles/well) were pre-incubated on eight empty wells blocked with TBS containing BSA (4%) and Tween-20 (0.1%) (TBST-BSA) to reduce non-specific binding and then transferred to the blocked target-coated wells for 1 h at 20°C. Plates were washed 20 times with TBS containing Tween-20 (0.1%) (TBST) and 20 times with TBS. Bound phages were then eluted, using either classical acidic conditions (0.1 M glycine pH 2.5 for 10 min at RT) or specific elution with the free target in solution at 10 μM for various times. The eluted phages were recovered to infect 5 mL of XL1-Blue cell suspension and plated onto large agar plates containing ampicillin (200 μg mL^−1^), tetracyclin (12.5 μg mL^−1^) and glucose (1%, w/v). The recovered bacteria were further used for the following selection round.

### Phage display selection on biotinylated EGFP

The gene coding for the target EGFP was sub-cloned into a modified pQE81L (Qiagen) vector in phase with the sequences coding for an N-terminal AviTag^TM^ (GLNDIFAQKIEWHE) followed by a TEV cleavage site (ENLYFQS). The plasmid was transformed into XL1-Blue cells previously transformed with pBirAcm (Avidity) allowing IPTG inductible biotin ligase expression. Cytoplasmic expression and biotinylation of the fusion protein were induced simultaneously by the addition of IPTG (1 mM) and biotin (100 μM). Cells were recovered in PBS (50 mM sodium phosphate pH 7.0, 150 mM NaCl), sonicated and centrifuged. The supernatant corresponding to bacterial soluble fraction was directly incubated in the streptavidin coated (10 μg mL^−1^) and blocked with TBST-BSA microtiter plate. Selection was performed as described above except for elution. Bound phages were specifically eluted upon incubation with TEV protease (10 μg mL^−1^) for 3 h at 25°C. Recovered phages were used for subsequent selection rounds.

### Screening for target binding by phage-ELISA

After three selection rounds, individual clones were screened for target binding by phage-ELISA essentially as previously described [33], [55]. Individual colonies were randomly picked and grown overnight at 37°C in a 96-well plate in 2YT (150 μL) containing ampicillin (200 μg mL^−1^), tetracyclin (12.5 μg mL^−1^) and glucose (1%, w/v). This master plate was used as a pre-culture plate for phage production and as a matrix stored at −80°C in the presence of glycerol (20%). Exponentially growing cells were infected for 1 h at 37°C with 10^10^ particles of helper phage and transferred into 2YT (1.5 mL) containing ampicillin (200 μg mL^−1^) and kanamycin (50 μg mL^−1^) in a deep–well culture plate. The phage particles were produced overnight at 30°C. A maxisorp ELISA plate (Nunc) was coated with the target (20 μg mL^−1^) in PBS overnight at 4°C. The plates were blocked with TBST-BSA for 3 h at 15°C, washed with TBST and 100 μL of the phage supernatant from each well were added and incubated for 2 h at 15°C. The plates were washed 4 times with TBST and bound phages were revealed with a horseradish peroxidase conjugated anti-M13 monoclonal antibody (Amersham) and detected at 450 nm using BM Blue POD as a substrate (Roche Diagnostic) after the addition of HCl. For each clone, a control with a non-coated well was performed on the same ELISA plate.

### Secondary screening for αRep expression and binding

For some targets, secondary screens were performed for positive clones to test the expression and binding capacities of the free soluble αRep. Soluble expression of αRep proteins was analyzed by CoFi blot or western blot experiments as previously described [32], [55]. Binding properties of the binders were qualitatively observed using ELISA experiments. Individual clones were grown at 37°C. Expression was induced by the addition of 1 mM IPTG and the cells were further incubated for 4 h. The bacteria were recovered at a normalized OD_600 nm_ and lysed with the B-PER^®^ reagent (Thermo Scientific) for 30 min at 37°C. The soluble fractions obtained after centrifugation were diluted 5 to 10 times and transferred on a previously target-coated and blocked ELISA plate. The presence of αRep proteins bound to the target was revealed using a horseradish peroxidase conjugated anti-flag^®^ M2 monoclonal antibody (Sigma). Clones screened as positive were further sequenced and the corresponding αRep genes were sub-cloned for αRep protein production and purification.

### Protein expression and purification

αRep variants and EGFP genes were sub-cloned respectively in the pQE31 and pQE81L vectors (Qagen). Expression and purification of αRep and GFP proteins were performed as described [32]. The plasmid coding for each protein was transformed into the expression *E. coli* strain M15 [pREP4] (Qiagen). Cells were grown at 37°C in 2YT medium containing 200 μg L^−1^ ampicillin and 25 μg L^−1^ kanamycin to an absorbance of 0.6 at 600 nm. Protein expression was induced by addition of IPTG to 1 mM and the cells were further incubated for 4 h at 37°C. The cells were harvested, suspended in TBS, submitted to three freezing/thawing cycles, treated with benzonase for 30 min and sonicated.

NCS-wt and NCS-3.24 were expressed from pHDiex vectors and purified as previously described [39]. Growth conditions were based on those previously developed [56], [57] Cells freshly transformed with the expression vector were grown for 48 h in 2YT medium containing ampicillin at 30°C, without induction. The culture medium was separated from the bacteria, and soluble proteins secreted into the culture medium were precipitated with 650 g of ammonium sulfate per liter. The proteins were collected by centrifugation. The precipitate was solubilized in TBS, dialyzed first against double-distilled water and then against 50 mM phosphate buffer, pH 8 containing 300 mM NaCl.

The coding sequence of Ebs1 N-terminal domain (hereafter named Ebs1) encompassing residues 1–610 was amplified from yeast Saccharomyces cerevisiae S288C genomic DNA and inserted into pET21-a vector with a sequence coding for a hexahistidine tag at the 3′ end of the gene, yielding plasmid pMG489. The protein was expressed in E. coli BL21 DE3 strain (Novagen) in 2YT medium supplemented with ampicillin (50 μg mL-1). At an absorbance at 600 nm of 0.8, the protein expression was induced at 37°C during 4 h by adding 1 mM IPTG. Cells were harvested by centrifugation and resuspended in 30 mL of Buffer A (20 mM Tris-HCl pH 7.5, 200 mM NaCl). Cell lysis was performed by sonication.

The His-tagged proteins were all purified from crude supernatant using nickel-affinity chromatography (Ni-NTA agarose, Qiagen) followed by size-exclusion chromatography (Hiload 16/60 Superdex^TM^ 75 or Superdex^TM^ 200 GE Healthcare) in PBS. For each protein, the purity of the final sample was checked by SDS–PAGE with an overloaded gel showing one well-resolved band with no visible contamination. For all the following experiments the proteins were quantified by UV spectrophometry and expressed in monomer concentration.

### Isothermal titration calorimetry

The binding parameters were monitored with an ITC 200 microcalorimeter (MicoCal). For the titration of target protein, 2 μL aliquots of the titrant (generally αRep binder) (200 to 350 μM depending on experiments) were injected from a computer-controlled 40 μL microsyringe at intervals of 180 s into the solution of target (20 to 35 μM; cell volume 0.24 μL) dissolved in the same standard buffer (PBS) while stirring at 1000 rpm. The heat of dilution of the binder was determined from the peaks measured after full saturation of target by the binder. The data were integrated to generate curves in which the areas under the injection peaks were plotted against the ratio of injected sample to cell content. Analysis of the data was performed using the MicroCal Origin^®^ software provided by the manufacturer according to the one-binding-site model. Changes in the free energy and entropy upon binding were calculated from determined equilibrium parameters using the equation: -RTLn(K_a_)  = ΔG° = ΔH° – TΔS°, where R is the universal gas constant (1.9872 cal mol^−1^ K^−1^), T is the temperature in Kelvin degrees, K_a_ is the association constant ΔG is the standard change in Gibbs free energy, ΔH° is the standard change in enthalpy and ΔS° is the standard change in entropy. The binding constant of each interaction is expressed as 1/K_a_ = K_d_ (in mol L^−1^).

#### Competition binding experiments assessed by ITC

The displacement titration of a mixture of A3 (25 μM) in presence of bA3-17 (43 μM) with bA3–2 (350 μM) has been performed in the same buffer as in all ITC experiments. Experimental data were fitted using the Origin^®^ software according to the competitive binding model based on the analysis of competition ligand binding experiment by displacement as described [58].

### Differential scanning calorimetry (DSC)

Thermal stability of proteins (0.1–0.9 mg mL^−1^ depending on the protein) in PBS was studied by differential scanning calorimetry (DSC) with a MicroCal VP-DSC instrument. Each measurement was preceded by a baseline scan with the buffer. Scans were done at 1 K min^−1^ between 20°C and 120°C. The heat capacity of the buffer was substracted from that of the protein sample before analysis. These corrected data were analyzed using a cubic spline as a baseline in the transition. Thermodynamic parameters ΔH_cal_ and ΔH_vH_ were determined by fitting the following equation to the data: ΔC_p_(T) = [K_d_(T) ΔH_cal_ ΔH_vH_]/[(1+ K_d_(T))^2^ RT^2^] where K_d_ is the equilibrium constant for a two-state process, ΔH_vH_ is the enthalpy calculated on the basis of a two-state process and ΔH_cal_ is the measured enthalpy.

### Analytical size-exclusion chromatography

Analytical SEC was done with an ÄKTA Purifier (GE Healthcare) system using a Superdex^TM^ 75 10/300 column (flow-rate 0.8 mL min^–1^) equilibrated in PBS. For all the purified proteins analyzed, 100 μL of protein sample (1–15 nmol depending on experiments) or 10 μL of final mixture of ITC experiment were injected onto the column. EachFor each elution profile, Abs_280 nm_ was normalized relatively to its maximum.

#### Affinity determination of bGFP-A with its target GFP by SPR

Surface Plasmon resonance was measured using a Biacore 2000 instrument. All measurements were performed in 10 mM HEPES, 150 mM NaCl, 3 mM EDTA, 0.005% Tween 20 at a flow rate of 50 μL min^−1^. Biotinylated GFP (2400 RU) was immobilized on a SA chip (GE Healthcare). For the determination of kinetics data, injections of bGFP-A were done during 120 seconds at different concentrations (71.3 nM to 1426 nM) and a final off-rate measurement of 10 minutes with buffer flow. The signal of an uncoated reference cell and buffer response was always substracted from the sensorgrams. The kinetic data of the interaction were evaluated by a separate fitting of k_off_ and k_on_ rates using the Origin^®^ software.

### Crystallization, structure determination and refinement

#### Complex A3/bA3–2

Bipyramidal crystals were obtained at 19°C from a 1∶1 mL mixture of a 400 μM protein complex solution (50 mM sodium phosphate pH 7; 150 mM NaCl) with a crystallization solution composed of 8% (w/v) PEG 2,000 MME, 100 mM Na acetate pH 4.6. For data collection, the crystals were cryo-protected by transfer into the crystallization solution with progressively higher ethylene glycol concentrations up to 30% (v/v) and then flash-cooled in liquid nitrogen. The diffraction data were recorded on beamline Proxima-1 (synchrotron SOLEIL, France). Data were processed using the XDS package [59]. The space group was P4_3_2_1_2 with one complex per asymmetric unit. The structure was determined by the molecular replacement method using our previously solved structure of the A3 protein (PDB code: 3LTJ [32]) as a template and the program MOLREP [60]. This model was further refined against the 1.9 Å resolution data with the ARP/WARP software [61], which automatically built most of the structure of the bA3–2 partner. This model was then refined using BUSTER [62] and rebuilt with COOT [63]. Regions 1–12 and 86–101 from bA3–2 as well as residues 1–6 and 197–202 from A3 protein are not visible in the density map and then absent from the final model, which contains residues 13 to 85 from bA3–2 and 7 to 196 from A3. In addition, 146 water molecules, 3 ethylene glycol molecules from the cryoprotection solution could be modelled into the electron density maps. The atomic coordinates (and structure factors) have been deposited into the Brookhaven Protein Data Bank under the accession numbers 4JW2.

#### Complex bNCS-16/NCS-3.24

Crystals of the NCS-3.24/bNCS-16 complex (380∶μM in 50∶mM sodium phosphate pH∶7; 150 mM NaCl) were obtained by mixing 1∶μL of complex solution with an equal volume of crystallization solution composed of 25% PEG 3350; 0.2∶M MgCl_2_; 0.1∶M BisTris pH∶5.5. Prior to data collection, crystals were cryo-protected using FOMBLIN Y LVAC 14/6 (Sigma-Aldrich) and flash-frozen in liquid nitrogen. The diffraction data were recorded on beamline Proxima-1 (synchrotron SOLEIL, France). Data were processed using the XDS package [59]. The space group was P2_1_2_1_2 with two heterodimeric complexes per asymmetric unit. The structure was determined by the molecular replacement method using our previously solved structure of the NCS-3.24 protein [39] and a model of the bNCS-16 (generated from the structure of A3 by removing two of the four internal repeats as well as the C-cap) as templates and the program MOLREP [60]. This model was then refined to 2.6 Å resolution using non-crystallographic symmetry restraints and TLS using BUSTER [62] and then rebuild with COOT [63]. Residues 1–7 for one bNCS-16 molecule and 1–8 for the second one as well as 105–139 from both bNCS-16 molecules are absent from the final model due to lack of electron density. Similarly, the amino terminal sequence encompassing the strep-tag (Ala-Trp-Ser-His-Pro-Gln-Phe-Glu-Lys-Ala-Ala) and the C-terminal residues encoding for the hexahistidine tag are absent in the final NCS-3.24 model because these are not defined in the density map. The final model also contains 6 water molecules. The atomic coordinates (and structure factors) have been deposited into the Brookhaven Protein Data Bank under the accession numbers 4JW3.

The statistics for data collection and refinement of both structures are summarized in Table 4. The complex interfaces were analyzed with the help of the PISA server [64].

**Table 4:** X-Ray data collection and refinement statistic.

	A3/bA3–2	NCS-3.24/bNCS-16
**Data statistics**		
Resolution (Å)	30–1.9 (1.95–1.9)	50–2.6 (2.76–2.6)
Space group	P4_3_2_1_2	P21212
Cell parameters	a = b = 87.7Å; c = 78Å	a = 118.2; b = 59.2Å; c = 65.5Å
Total number of reflections	135,887	55,290
Total number of unique reflections	24,498	14,080
Rsym (%) a	4.8 (56.6)	5.7 (55)
Completeness (%)	99.1 (98.9)	95.8 (96.3)
I/σ/I)	17.6 (2.)	15.7 (2.6)
Redundancy	5.5	3.9
**Refinement**		
Resolution (Å)	30–1.9	34–2.6
R/R_free_ (%) b	19.4/22.4	22.4/26.3
R.m.s.d. bonds (Å)	0.010	0.010
R.m.s.d. angles (°)	1.02	1.12
**Ramachandran plot**		
Most favoured (%)	97.4	95.2
Allowed (%)	2.6	4.8
**PDB code**	4JW2	4JW3

Values in parentheses are for highest resolution shell.

a R_sym_ = y_h_∑_i_|I_hi_ – <I_h_>|/-_h_∑_i_I_hi_, were I_hi_ is the *i*th observation of the reflection h, while <I_h_> is the mean intensity of reflection h.

b R_factor_  = ctorF_o_| – |F_c_||/|F_o_|. R_free_ was calculated with a small fraction (5%) of randomly selected reflexions.

### Accession numbers

The atomic coordinates have been deposited into the Brookhaven Protein Data Bank with accession numbers 4JW2 for the A3/bA3–2 complex and 4JW3 for the NCS-3.24/bNCS-16 complex.
